# A Rare Complication following Thyroid Percutaneous Ethanol Injection: Plummer Adenoma

**DOI:** 10.1155/2017/1026139

**Published:** 2017-03-29

**Authors:** Roberto Cesareo, Anda Mihaela Naciu, Valerio Pasqualini, Giuseppe Pelle, Silvia Manfrini, Gaia Tabacco, Angelo Lauria Pantano, Alessandro Casini, Roberto Cianni, Andrea Palermo

**Affiliations:** ^1^Department of Internal Medicine, “S. M. Goretti” Hospital, Latina, Italy; ^2^Department of Endocrinology, University Campus Bio-Medico, Rome, Italy; ^3^Department of Radiology, “S. M. Goretti” Hospital, Latina, Italy

## Abstract

Percutaneous ethanol injection (PEI) is a technique used only for benign thyroid nodules, cystic or mixed cystic-solid with a large fluid component. It is a quite low-cost, safe, and outpatient method of treatment. Rare and severe complications have been described after PEI: jugular vein thrombosis and severe ethanol toxic necrosis of the larynx combined with necrotic dermatitis. Moreover, only four thyrotoxicosis cases due to Graves' disease have been reported. We report a case of 58-year-old female with a voluminous thyroid cystic nodule, occupying almost the entire left thyroid lobe. Our patient had already performed surgical visit and intervention of thyroidectomy had been proposed to her, which she refused. At baseline, our patient has a normal thyroid function with negative autoantibodies. According to the nodular structure, intervention of PEI has been performed with a significant improvement of compressive symptoms and cosmetic disorders. About 30 days after treatment, there was a significant volume reduction, but patient developed an acclaimed symptomatic thyrotoxicosis. After ruling out several causes of hyperthyroidism and according to the thyroid scintigraphy findings, we made the diagnosis of Plummer adenoma. To our knowledge, our patient is the first case of Plummer adenoma following PEI treatment of nontoxic thyroid nodule.

## 1. Introduction

Thyroid nodules disease is one of the most common clinical endocrine disorders, mainly in iodine lacking regions. Thyroid nodules are characterized by undue growth of structure, functional transformation, and/or cystic degeneration of one or more zones inside the gland. Thyroid nodules are frequently incidental findings, following noninvasive methods such as thyroid ultrasound or radionuclide thyroid scans [1–4].

The progression and management of thyroid nodules are still controversial. If surgery is refused or contraindicated, there are currently a lot of alternative approaches including radioiodine treatment, levothyroxine therapy, percutaneous laser ablation (PLA), percutaneous radiofrequency ablation (RFA), and percutaneous ethanol injection (PEI) [5].

PEI under ultrasonography guidance has been used for more than ten years in solitary hot, toxic, and even cold thyroid nodules, but the most convincing effect was seen in solitary thyroid cysts. Presently, PEI is used only for benign thyroid nodules and cystic or mixed cystic-solids with large fluid components [5, 6]. PEI is quite a low-cost, safe, and outpatient method of treatment.

However, like other mini-invasive procedures, PEI may have partial efficacy, possible adverse effects, and, typically, mild complications [5]. In the related literature, cases of thyrotoxicosis due to Graves' disease have been identified subsequent to PEI. To our knowledge, our case is the first thyrotoxicosis caused by Plummer's adenoma following treatment with PEI.

## 2. Case Presentation

**Figure 1:**
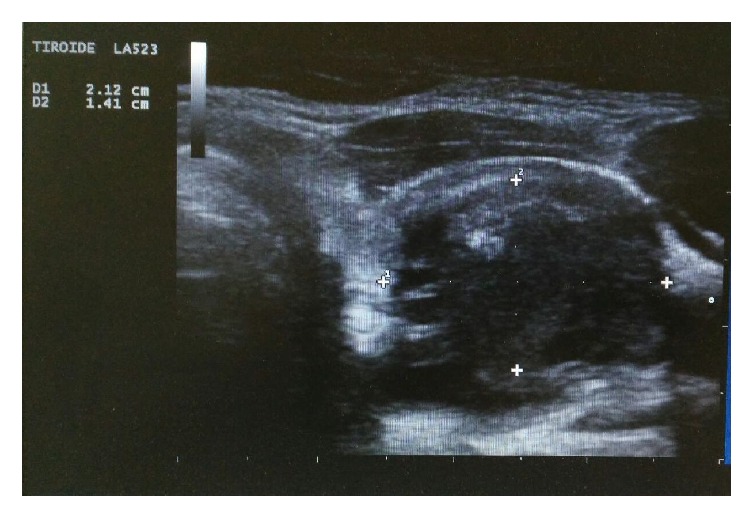
Left side nodule before PEI.

We report a case of a 58-year-old female presented to our center with a voluminous thyroid nodule overall size of 40 × 33 × 26 mm (volume of 17.9 ml), occupying almost the entire left thyroid lobe (Figure 1). Our patient had already had a surgical visit and an intervention of thyroidectomy had been proposed to her, which she refused. A thyroid function test demonstrated TSH 0.8 mIU/l, FT3: 3.77 pg/ml, and FT4: 1.37 ng/dL (normal values for TSH: 0.3–3.74 mIU/l; FT3: 2.2–4.2 pg/ml, and FT4: 0.8–1.7 ng/dL), peroxidase antibodies (TPOAb) 31.2 IU/ml and thyroglobulin antibodies (TgAb) 18 IU/ml (normal values for TPOAb: 0–60 UI/ml; TgAb: 0–60 UI/ml). After evaluation, symptom score and cosmetic score were positive. A thyroid ultrasound demonstrated a right lobe with a normal volume and a left lobe with increased volume (right volume 6.1 ml, left volume 22.2 ml), with normal echogenicity and moderately inhomogeneous echotexture. Due to the nodular structure, intervention of PEI was proposed to which she expressed favorable consensus.

Before PEI, our patient had been submitted to fine needle aspiration on this mixed cystic-solid thyroid nodule, with a cytological diagnosis of Thy2: nonneoplastic. With the patient in a supine position, a total dose of 5 ml of 95% sterile ethanol was injected slowly via a 22 gauge needle under real time ultrasound guidance [7]. We monitored the injection as a hyperechogenic region and completed the procedure in five minutes. After a successful PEI procedure, color Doppler examination showed complete disappearance of intranodular hypervascularization.

**Figure 2:**
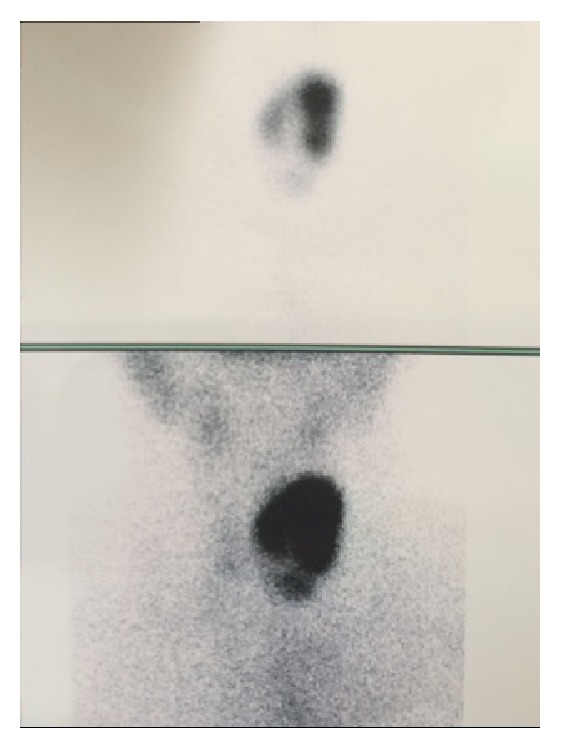
Thyroid technetium 99m scintigraphy showing hyperactive nodule on the left lobe and full suppression in the remainder of the gland.

Following PEI treatment, the patient detected immediate improvement of compressive symptoms and cosmetic disorders. About 30 days after the treatment there was a significant volume reduction (5.9 ml versus baseline volume of 17.9 ml, volume reduction rate of 67%) but the patient complained of dyspnea, tremors and tachycardia. The cardiorespiratory function parameters were as follows: blood pressure 150/85 mmHg, heart rate 112/min with an ECG highlighted sinus tachycardia, breath frequency 20/min, and oxygen saturation 96%. Thyroid hormone tests showed a framework of acclaimed thyrotoxicosis TSH < 0.01 mIU/l; FT3: 5.8 pg/ml, FT4 19.1 ng/dL (normal values for TSH: 0.3–3.74 mIU/l; FT3: 2.2–4.2 pg/ml; and FT4: 0.8–1.7 ng/dL). Autoantibodies measurements were performed (TRAb, TPOAb, and TgAb) to investigate the cause of thyrotoxicosis. These autoantibodies were undetectable and we ruled out the diagnosis of Graves' disease and hashitoxicosis. The lack of neck pain and the normality of acute inflammation parameters (erythrocyte sedimentation rate (ESR) and PCR) allowed us to exclude the diagnosis of subacute thyroiditis. The patient was therefore submitted to thyroid scintigraphy which showed a left nodule under complete functional autonomy, corresponding to the nodule treated with PEI (Figure 2). Based upon clinical, laboratory, and imagistic findings, a diagnosis of Plummer adenoma was made.* After thyrostatic treatment (methimazole 15 mg/day for 3 months) and beta-blockers (Propranolol 40 mg 1 cpr every 8 hours for 3 months), the patient has been treated with radioiodine therapy (131I), achieving a euthyroid status.*

## 3. Discussion

PEI is a nonsurgical option of therapy for cystic or mixed cystic-solid benign thyroid nodules, with an important quote of liquid, with efficacy ranging from 38 to 85% [8]. It was proposed for the first time by Livraghi et al., for treating autonomously functioning thyroid nodules [9], but is no longer used for solid nodules. In addition to its favorable outcomes, PEI has the benefit of not affecting extranodular thyroid tissue as occurs when using I131 therapy, which exposes the surrounding thyroid tissue to significant radiation doses [7].

Injection of ethanol causes reduction in the volume of cystic thyroid nodules by causing cellular dehydration and protein denaturation, leading to reactive fibrosis, as documented in a few papers with thyroid histopathology following PEI treatment [10]. A histopathological investigation after an intranodular ethanol injection shows local injury associated with small vessels thrombosis, a complex and irreversible hemorrhagic infarction, coagulative necrosis, and fibrosis on areas outside the nodule [11]. PEI is a relatively safe technique, well-tolerated, effective, and inexpensive [12], with common side effects such as local pain that may radiate to the jaw or retroauricular area, transient dysphonia, flushing, dizziness, fever lasting a day, and hematoma.

In the related literature, severe but rare complications have been described after PEI, such as jugular vein thrombosis and severe ethanol toxic necrosis of the larynx combined with necrotic dermatitis [13]. Moreover, three cases of Graves' disease without Graves' ophthalmopathy (after treatment of toxic thyroid adenomas) and one case of Graves' disease with severe Graves' ophthalmopathy (after treatment of mixed cystic-solid, nontoxic thyroid nodule) have been also reported [5].

Graves' disease is a complication that can be expected after PEI, probably due to the extensively damaged follicular thyroid cells. The mechanism for causality between the PEI and Graves' disease is not completely known. Regalbuto et al. issued a theory that contended that the destruction of thyroid tissue after injection of ethanol, among subjects genetically predisposed to autoimmune reactions, could release a large quantity of antigenic material (including TSHr protein) from follicular thyroid cells that may trigger an autoimmune inflammatory response throughout thyroid and orbital soft tissues [5].

Usually, there is a transient elevation in serum concentration of thyroid hormones resulting in the sudden release of stored thyroid hormones after follicular destruction produced by an ethanol injection.

This phenomenon does not lead to any clinical and biochemical consequences in nontoxic nodules because free thyroid hormones' concentrations are constantly within limits. Among patients with toxic nodules, a moderate worsening of symptoms could appear [14].

Our patient was a female with no family history of autoimmune disease, with TSH and free thyroid hormones within limits and with no clinical signs of hyperthyroidism before PEI, even if her TSH was at the lower limit of the normal range. All autoantibodies were measured before and after PEI, with normal values, which allowed us to rule out the theory speculated by Regalbuto et al. Unfortunately, thyroid scintigraphy before PEI was not performed because there was no reason in the absence of any signs or symptoms. Therefore, a preexistent form of subclinical Plummer adenoma cannot be ruled out.

To our knowledge, our patient is the first case of Plummer adenoma following PEI treatment of nontoxic thyroid nodule.

## 4. Conclusions

In spite of the fact that severe complications after PEI treatment are rare, they should be considered in subjects who have indication to this kind of treatment.
